# Draft Genome Sequence of *Plasmopara viticola*, the Grapevine Downy Mildew Pathogen

**DOI:** 10.1128/genomeA.00987-16

**Published:** 2016-09-22

**Authors:** Yann Dussert, Jérôme Gouzy, Sylvie Richart-Cervera, Isabelle D. Mazet, Laurent Delière, Carole Couture, Ludovic Legrand, Marie-Christine Piron, Pere Mestre, François Delmotte

**Affiliations:** a SAVE, Bordeaux Sciences Agro, INRA, Villenave d'Ornon, France; b LIPM, Université de Toulouse, INRA, CNRS, Castanet-Tolosan, France; c SVQV, INRA, Université de Strasbourg, Colmar, France

## Abstract

*Plasmopara viticola* is a biotrophic pathogenic oomycete responsible for grapevine downy mildew. We present here the first draft of the *P. viticola* genome. Analysis of this sequence will help in understanding plant-pathogen interactions in oomycetes, especially pathogen host specialization and adaptation to host resistance.

## GENOME ANNOUNCEMENT

*Plasmopara viticola* is a heterothallic diploid oomycete (*Stramenopiles*) responsible for grapevine downy mildew, one of the most serious grapevine diseases worldwide (1, 2). The pathogen is native to North America and was accidentally introduced in Europe at the end of the 19th century (3). *P. viticola* is a good candidate for the study of host adaptation in biotrophic plant pathogens, especially host-plant specialization (4), and adaptation to plant resistance (5, 6). Analyses of transcriptomic data for the species have recently been published (7, 8), but until now, no reference genome sequence was available.

The sequenced isolate INRA-PV221 was collected in 2009 from a grapevine leaf lesion in a vineyard in the Bordeaux region (Blanquefort, France). The isolate was propagated on detached grapevine leaves, and genomic DNA of sporangia was extracted using either the DNeasy blood and tissue kit or the DNeasy plant minikit (both from Qiagen). A paired-end and two mate-pair libraries (3- and 8-kb inserts) were prepared and sequenced on Illumina HiSeq2000 sequencers at the GeT-PlaGe GenoToul facility (Toulouse, France) and by Eurofins MWG Operon (Ebersberg, Germany), respectively, producing around 615 million reads. Paired-end reads were cleaned and transformed into virtual long reads using boost-r (J. Gouzy, unpublished data), and then assembled into contigs using Velvet (9). After removal of contigs included in longer contigs, scaffolding of contigs using mate-pair read information was carried out with LYNX (J. Gouzy, unpublished data).

After removing contaminating bacterial sequences, the assembly included 1,883 scaffolds with a size greater than 1 kb (maximum size: 763.2 kb), for a total size of 74.74 Mb (N content: 2.12 Mb). The *N_50_* and *N_90_* of the assembly were 180.6 kb and 33.5 kb, respectively (*L*_50_: 130 scaffolds; *L*_90_: 450 scaffolds). The average GC content was 44.3%. Completeness of the genome as estimated by CEGMA (10, 11), using a set of 248 conserved eukaryote genes, was 91% (95% when counting partial matches).

The gene annotation and analysis of the genome of *P. viticola* will lead to the identification of the effector gene repertoire of this pathogen, allowing a better understanding of the molecular interactions governing this pathosystem and facilitating the identification of genes involved in gene-for-gene interactions with grapevine. The availability of a reference genome will also be helpful for population genomics studies addressing the worldwide invasion of *P. viticola* and the mechanisms responsible for its rapid adaptation to fungicides and to resistant grapevine cultivars (5, 6).

### Accession number(s).

This whole-genome shotgun project has been deposited in DDBJ/ENA/GenBank under the accession number MBPM00000000. The version described in this paper is the first version, MBPM01000000.

## References

[1] Kamoun S, Furzer O, Jones JD, Judelson HS, Ali GS, Dalio RJ, Roy SG, Schena L, Zambounis A, Panabières F, Cahill D, Ruocco M, Figueiredo A, Chen X-R, Hulvey J, Stam R, Lamour K, Gijzen M, Tyler BM, Grünwald NJ, Mukhtar MS, Tomé DFA, Tör M, Van Den Ackerveken G, McDowell J, Daayf F, Fry WE, Lindqvist-Kreuze H, Meijer HJG, Petre B, Ristaino J, Yoshida K, Birch PRJ, Govers F 2015 The top 10 oomycete pathogens in molecular plant pathology. Mol Plant Pathol 16:413–434. doi:10.1111/mpp.12190. 25178392 PMC6638381

[2] Gessler C, Pertot I, Perazzolli M 2011 *Plasmopara viticola*: a review of knowledge on downy mildew of grapevine and effective disease management. Phytopathol Mediterr 50:3–44.

[3] Fontaine MC, Austerlitz F, Giraud T, Labbé F, Papura D, Richard-Cervera S, Delmotte F 2013 Genetic signature of a range expansion and leap-frog event after the recent invasion of Europe by the grapevine downy mildew pathogen *Plasmopara viticola*. Mol Ecol 22:2771–2786. doi:10.1111/mec.12293. 23506060

[4] Rouxel M, Mestre P, Comont G, Lehman BL, Schilder A, Delmotte F 2013 Phylogenetic and experimental evidence for host-specialized cryptic species in a biotrophic oomycete. New Phytol 197:251–263. doi:10.1111/nph.12016. 23153246

[5] Delmotte F, Mestre P, Schneider C, Kassemeyer H-H, Kozma P, Richart-Cervera S, Rouxel M, Delière L 2014 Rapid and multiregional adaptation to host partial resistance in a plant pathogenic oomycete: evidence from European populations of *Plasmopara viticola*, the causal agent of grapevine downy mildew. Infect Genet Evol 27:500–508. doi:10.1016/j.meegid.2013.10.017. 24184095

[6] Delmas CE, Fabre F, Jolivet J, Mazet ID, Richart Cervera S, Delière L, Delmotte F 2016 Adaptation of a plant pathogen to partial host resistance: selection for greater aggressiveness in grapevine downy mildew. Evol Appl 9:709–725. doi:10.1111/eva.12368. 27247621 PMC4869412

[7] Mestre P, Carrere S, Gouzy J, Piron M-C, Tourvieille de Labrouhe D, Vincourt P, Delmotte F, Godiard L 2016 Comparative analysis of expressed CRN and RXLR effectors from two *Plasmopara* species causing grapevine and sunflower downy mildew. Plant Pathol 65:767–781. doi:10.1111/ppa.12469.

[8] Yin L, Li X, Xiang J, Qu J, Zhang Y, Dry IB, Lu J 2015 Characterization of the secretome of *Plasmopara viticola* by *de novo* transcriptome analysis. Physiol Mol Plant Pathol 91:1–10. doi:10.1016/j.pmpp.2015.05.002.

[9] Zerbino DR, Birney E 2008 Velvet: algorithms for *de novo* short read assembly using de Bruijn graphs. Genome Res 18:821–829. doi:10.1101/gr.074492.107. 18349386 PMC2336801

[10] Parra G, Bradnam K, Korf I 2007 CEGMA: a pipeline to accurately annotate core genes in eukaryotic genomes. Bioinformatics 23:1061–1067. doi:10.1093/bioinformatics/btm071. 17332020

[11] Parra G, Bradnam K, Ning Z, Keane T, Korf I 2009 Assessing the gene space in draft genomes. Nucleic Acids Res 37:289–297. doi:10.1093/nar/gkn916. 19042974 PMC2615622

